# Developing confidence in basic prescribing skills during medical school: a longitudinal questionnaire study investigating the effects of a modified clinical pharmacology course

**DOI:** 10.1007/s00228-018-2508-3

**Published:** 2018-06-28

**Authors:** Anna L. Eriksson, Susanna M. Wallerstedt

**Affiliations:** 1 Department of Internal Medicine and Clinical Nutrition, Sahlgrenska Academy, University of Gothenburg, 413 45 Gothenburg, Sweden; 2 Department of Clinical Pharmacology, Sahlgrenska University Hospital, Gothenburg, Sweden; 3 Department of Pharmacology, Sahlgrenska Academy, University of Gothenburg, Gothenburg, Sweden

**Keywords:** Clinical pharmacology, Medical school, Medication review, Practice, Prescribing

## Abstract

**Purpose:**

To investigate if increased focus on pharmacotherapy during medical school can increase students’ confidence in basic prescribing skills, that is, performing medication reviews and writing medication discharge summaries.

**Methods:**

In 2016, the clinical pharmacology course in medical school in Gothenburg, Sweden, was modified to facilitate the students’ acquisition of prescribing skills, with (i) clarified learning outcomes; (ii) supply of a list of common drugs for self-completion; (iii) instructions to practice medication reviews/discharge summaries during the ward-based education; and (iv) a concluding compulsory seminar where the students were to present prescribing-related experiences from their ward-based attendance. Questionnaires were administered to students participating in the course before (2016; *n* = 101) and after (2017; *n* = 137) implementation of the modifications. Students were asked to grade their agreement from 1 (totally disagree) to 5 (totally agree) on statements related to their perceived confidence in basic prescribing skills.

**Results:**

In all, 195 students returned the questionnaire (response rate 82%; median age 24 years; 68% female). Confidence was rated higher after the modifications were implemented, both regarding medication reviews and medication discharge summaries, after vs. before 3.6 ± 1.2 vs. 3.2 ± 1.0 (*P* = 0.024), and 4.3 ± 0.9 vs. 3.9 ± 1.1 (*P* = 0.008), respectively. The adjusted odds for being confident in performing these tasks were 1.49/1.36 times greater after the course modifications (*P* = 0.047/0.019). Perceived confidence in performing medication reviews/summary reports was positively correlated with numbers performed (*P* < 0.0001).

**Conclusions:**

Modifications of the clinical pharmacology course during medical school, focusing on students’ training in pharmacotherapy, was associated with increased confidence of this core skill for a physician.

**Electronic supplementary material:**

The online version of this article (10.1007/s00228-018-2508-3) contains supplementary material, which is available to authorized users.

## Introduction

Prescribing is a core skill for a physician. Therefore, and for rational and safe use of medicines, it is essential that the curricula of medical schools are constructed to ensure that graduates have acquired appropriate prescribing competencies including knowledge, skills, and attitudes [1]. However, concerns have been raised that medical schools do not prepare students to a sufficient extent in the art of prescribing [2–5]. In this context, the clinical pharmacology undergraduate course may contribute; an essential task for clinical pharmacologists is to teach medical students and to facilitate their learning in the area of pharmacotherapy [6–8].

In the curriculum of medical school in Gothenburg, Sweden, clinical pharmacology is taught during the sixth and seventh terms. In all, the clinical pharmacology course encompasses 5 days of lectures and seminars, focused on general pharmacotherapeutic aspects such as pharmacokinetics, adverse drug reactions, and interactions. The course also includes small group discussions on pharmacotherapy in fictive patient cases, with eight to ten students supervised by a specialist or a resident in clinical pharmacology.

The course in clinical pharmacology is integrated in the internal medicine course, the latter including ward-based education. This arrangement provides ample opportunities for the students to practice basic prescribing skills in real patients, that is, performing medication reviews and writing medication discharge summaries. Indeed, training under real-life circumstances during medical school may enhance learning [9–11]. Medication reviews, regulated in statutes from the Swedish National Board of Health and Welfare [12], entail that the physician reconciles the drug treatment and ascertains that it is reasonable given the patient’s health status. It includes, for example, considering side effects as a possible cause of the symptoms; reflecting on the importance of renal function for a specific drug; determining the clinical relevance of potential drug-drug interactions, with appropriate treatment adjustments where needed; and providing relevant information to the patient and the next caregiver at discharge in a medication discharge summary.

Although the ward-based education provides opportunities for medical students to practice medication reviews and discharge summaries, all students may not take advantage of these opportunities. Therefore, the ward-based education per se may not be sufficient for students to develop basic prescribing skills. Indeed, students focus their efforts to pass the examination [13]. Therefore, to stimulate learning within the art of prescribing, it may be preferable with clear instructions including learning outcomes and an examination. We performed this study to investigate if a modified clinical pharmacology course during medical school could increase the students’ confidence in performing medication reviews and writing medication discharge summaries.

## Methods

In 2016, we implemented modifications in the clinical pharmacology course for medical students to facilitate their acquisition of knowledge and skills related to prescribing. Four course-related aspects were targeted. We anchored the modifications with the course leaders and tutors of the internal medicine course, which is taught in parallel.

First, we revised the learning outcomes, clearly stating that upon completion of the course, students shall demonstrate knowledge of common internal medicine drugs (Appendix 1). In addition, the revised learning outcomes clarified that the students shall be able to perform medication reviews and to write medication discharge summaries.

Second, as a means to facilitate learning, we supplied the students with a list of common drugs, consisting of about 200 substances arranged in therapeutic areas, for self-completion regarding brand names, mechanisms of action, indications, common doses, and whether the substance is recommended in regional prescribing guidelines (Appendix 2). We identified relevant substances in the guidelines booklet [14], and by a top 200 search in the aggregated register on dispensed drugs from pharmacies in Sweden (Concise).

Third, we instructed the students to perform medication reviews and medication discharge summaries during their ward-based education, according to the instructions by the Swedish National Board of Health and Welfare [12] and presented in a quick reference guide (Appendix 3). These physician tasks were explained and further exemplified using patient cases during the first lecture in the clinical pharmacology course. National indicators of prescribing quality were discussed as a means to get a quick guide to drug treatment which in general is considered appropriate in the elderly [15], as well as their limitations regarding applicability for a specific patient [16, 17]. During routine teacher meetings, we presented the changes of the course and involved the clinical tutors to ascertain that the students were supervised and supported during their training. In addition, for a student to pass the ward-based part of the internal medicine course, the clinical tutor made an overall judgment of the student’s performance in the ward. In this assessment, medication reviews/discharge summaries were natural parts as drug treatment is a central component of patient care in this specialty.

Fourth, we instructed the students to collect and discuss patient cases from their ward-based education, from a pharmacotherapeutic perspective, in student groups of ten. They were also instructed to prepare a presentation for their fellow students in a 3-h concluding and compulsory seminar (3–4 groups per occasion) supervised by teachers in clinical pharmacology. The students were free to focus on any aspect within pharmacotherapy and no specific format was provided for the presentations.

The list of common drugs and the learning outcomes, as well as the instructions to perform medication reviews/discharge summaries and to prepare for the concluding seminars, were all uploaded to the Learning Management System, e-mailed to the students, and communicated during the clinical pharmacology lectures.

To evaluate the effects of the modified course on the students’ perceived confidence in basic prescribing skills, we distributed anonymous questionnaires to all students after the completion of the clinical pharmacology course, before (2016; *n* = 101, paper questionnaire) and after (2017; *n* = 137, electronic questionnaire) the course was modified (Appendix 4). The questionnaire included background information of the respondent (age, sex, research experience categorized as any or none, number of medication reviews/discharge summaries performed) as well as Likert questions where the students were asked to grade their agreement from 1 (totally disagree) to 5 (totally agree) on statements regarding their confidence in basic prescribing skills, as well as the extent of their reflections regarding important prescribing aspects during the ward-based education. The questionnaire was developed by a team of clinical pharmacologists and tested for face validity on colleagues.

According to Swedish regulations, ethics approval is not required in anonymous questionnaire studies. The students were informed orally at a compulsory seminar, and in writing, that the aim of the questionnaire was to improve medical school to facilitate students’ acquisition of prescribing knowledge and skills and that participation was anonymous and voluntary.

### Statistics

The statistical analyses were performed with SPSS (IBM SPSS Statistics for Windows, Version 23.0, Armonk, NY). We used the Mann Whitney and the chi-square tests for comparisons between groups. Before implementation of the course modifications, half of the students completed the questionnaire when 2 weeks remained of the ward-based education. Therefore, the comparison statistics (before versus after) were made using the subgroup of before participants that had completed all ward-based education. Results are presented as median (interquartile range). To facilitate interpretation, mean ± standard deviation is also presented where appropriate, although normal distribution was not assumed in the statistical analyses. In dichotomized analyses, we categorized students responding 4 or 5 on Likert questions as confident in performing medication reviews/discharge summaries, and as reflecting on various aspects of drug treatment. To investigate the importance of the implemented modifications for the students’ perceived confidence regarding basic prescribing skills (performing medication reviews and medication discharge summaries, respectively), we performed logistic regression analyses resulting in crude and adjusted odds ratios with 95% confidence intervals (CI). Age (continuous variable), sex (female vs. male), and research experience (any vs. none) were included as covariates. We also calculated Spearman’s correlation coefficients between the number of performed medication reviews/discharge summaries and the perceived professional confidence, as well as the extent of reflection on important prescribing aspects.

## Results

In 2016, before the course in clinical pharmacology was modified, 94 (93%) out of 101 students returned the questionnaire. Among these, 49 (out of 51) had completed all ward-based education. In 2017, after the course was modified, 101 (74%) out of 137 students returned the questionnaire. In all, 195 students returned the questionnaire (response rate 82%). Characteristics of respondents did not differ significantly between the groups (Table 1). The students had a mean age of 24 years, ranging from 21 to 48 years, 126 (68%) were women, and 39 (20%) reported research experience.

**Table 1:** Characteristics of responding medical students before and after the modified course in clinical pharmacology. Values are presented as counts/number of respondents (percentage) or median (interquartile range)

	Before		After	*P* value Before (subgruoup) vs after
	All *n* = 94	Subgroup* n = 49	All *n* = 101	
Female sex	63/94 (67)	29/49 (59)	63/101 (62)	0.71
Age	24 (23–27)	24 (23–26)	24 (22–26)	0.56
Research	13/82 (16)	8/43 (19)	26/100 (26)	0.34

*Students who at the time of the questionnaire had performed all ward-based education in the internal medicine course

In all, 88 (45%) students were confident in performing medication reviews, and 136 (70%) in writing medication discharge summaries. The students reported higher confidence in basic prescribing skills after the course was modified, both concerning performing medication reviews and writing medication discharge summaries (Table 2). The crude and adjusted odds ratios for the modified course to predict confidence were 1.42 (95% CI 1.09; 1.90; *P* = 0.016) and 1.36 (1.00; 1.84; *P* = 0.047) for medication reviews, and 1.53 (1.16; 2.09; *P* = 0.008) and 1.49 (1.07; 2.07; *P* = 0.019) for medication discharge summaries.

**Table 2:** Students’ agreement with statements regarding their confidence in the basics of prescribing that is performing medication reviews and writing medication discharge summaries, ranging from 1 (completely disagree) to 5 (completely agree), before and after the clinical pharmacology course was modified

		Before		After	*P* value Before (subgroup) vs. after
		All *n* = 94	Subgroup *n* = 49	All *n* = 101	
I feel confident performing a medication review	Median (IQR)	3 (2–4)	3 (2–4)	4 (3–5)	0.024
	Mean ± SD	3.0 ± 1.0	3.2 ± 1.0	3.6 ± 1.2	
	Confident*	34 (36)	19 (39)	54 (53)	
I feel confident writing a medication discharge summary	Median (IQR)	4 (3–4.25)	4 (3–5)	5 (4–5)	0.008
	Mean ± SD	3.6 ± 1.2	3.9 ± 1.1	4.3 ± 0.9	
	Confident*	57 (61)	35 (71)	79 (78)	

IQR, interquartile range; SD, standard deviation

*Rating 4 or 5

The extent of practice of medication reviews and medication discharge summaries was correlated with perceived confidence (Table 3). Of 47 (24%) students having performed 0–1 medication reviews, 10 (21%) were confident in performing the task. Of 56 (28%) students having performed ≥ 6 medication reviews, 36 (64%) were confident. Correspondingly, of 33 (17%) students having written 0–1 medication discharge summaries, 12 (36%) were confident in performing the task. Of 77 (39%) students having written ≥ 6 medication discharge summaries, 68 (88%) were confident.

**Table 3:** Number of medical students (percent) being confident* in performing medication reviews and writing medication discharge summaries by the numbers performed, and correlation between these aspects by Spearman

	Number of medication reviews/discharge summaries performed, with the number of students having performed the corresponding numbers within parentheses				Correlation	
	0–1 (*n* = 47/33)	2–3 (*n* = 41/31)	4–5 (*n* = 49/52)	≥ 6 (*n* = 56/77)	Coefficient	*P* value
Medication review	10 (21)	14 (34)	27 (55)	36 (64)	0.44	< 0.0001
Medication discharge summary	12 (36)	18 (58)	37 (71)	68 (88)	0.50	< 0.0001

*Rating 4 or 5 on statements that they feel confident, ranging from 1 (completely disagree) to 5 (completely agree)

Regarding the students’ reflections on important drug treatment aspects, most students reflected on what drugs their patients were prescribed (Table 4). About half of the students reflected on actual medication intake, reasonableness, adverse drug reactions, double medication, and renal function, and fewer than half reflected on interactions and dosing. Investigating the correlation between the number of medication reviews performed and the reflection on important drug treatment aspects in the whole cohort, we found a positive and significant correlation, with correlation coefficients between 0.17 and 0.38 for aspects like medication reconciliation, reasonableness of the drug treatment, potential double medication, dosing, renal function, and interactions.

**Table 4:** Number of medical students (percent) who agreed* to statements regarding their reflection on important drug treatment aspects during the ward-based education, summarized and by the number of medical reviews performed, and correlation between these aspects by Spearman

		All (*n* = 195)	According to number of medication reviews, with the number of students having performed the corresponding numbers within parentheses				Correlation	
			0–1 (*n* = 47)	2–3 (*n* = 41)	4–5 (*n* = 49)	≥ 6 (*n* = 56)	Coefficient	*P* value
During the ward-based education, I usually reflected on…	…what drugs my patients were ordered	155 (79)	34 (72)	27 (66)	41 (84)	52 (93)	0.33	< 0.001
	…what drugs my patients were actually using	92 (47)	11 (23)	20 (49)	22 (45)	38 (68)	0.38	< 0.001
	…whether the drug treatment was reasonable given the patient’s condition	116 (59)	20 (43)	23 (56)	31 (63)	40 (71)	0.27	< 0.001
	...whether adverse reactions could cause the patient’s symptoms	98 (50)	22 (47)	19 (46)	23 (47)	33 (59)	0.08	0.29
	…that double medication shall be avoided	110 (56)	22 (47)	20 (49)	33 (67)	35 (62)	0.22	0.002
	…whether the dose was reasonable	37 (19)	4 (9)	7 (17)	10 (20)	16 (29)	0.31	< 0.001
	…the importance of the patient’s kidney function for the drug treatment	97 (50)	18 (38)	19 (46)	27 (55)	32 (57)	0.17	0.018
	…whether there were drug-drug interaction	66 (34)	11 (23)	11 (27)	18 (37)	25 (45)	0.28	< 0.001

*Rating 4 or 5 on statements, with responses ranging from 1 (completely disagree) to 5 (completely agree)

## Discussion

In this study, we show that medical students’ acquisition of confidence in basic prescribing skills may be facilitated by a modified course in clinical pharmacology, integrated in the internal medicine course and clearly focusing on students to practice medication reviews and medication discharge summaries during their ward-based education. Indeed, relatively small modifications, including clarified learning goals, supply of a list of common drugs, clear “how-to-do” instructions, and an examining seminar were associated with 40–50% increased odds for the students to be confident in these core aspects within the professional role of a physician. After the modification of the course, more than half of the students were confident in performing medication reviews and almost eight in ten students in writing medication discharge summaries. Upon practicing medication reviews and medication discharge summaries, the students also reflected to a greater extent on important drug treatment aspects.

Our results highlight the importance of training for gaining professional confidence in prescribing. Indeed, the need to educate medical students regarding medication reviews has been discussed previously [18]. Undoubtedly, it takes considerable time and efforts to become a skilled physician mastering the art of prescribing. This process must be entered during medical school; drug treatment is a complex task where diagnostic competence needs to be combined with pharmacological knowledge and patient communication. For rational use of medicines, physicians also need pharmacotherapy skills and sound attitudes towards prescribing. In fact, an overemphasis on algorithmic rules may make health care less patient-centered, and evidence-based guidelines often map poorly to complex multimorbidity [19]. Our results are supported by a recent study in which medical students were found to prefer hands-on participation rather than observation to build professional confidence [20]. Further, student-run clinical consultations have been found valuable [11, 21].

The association between the number of medication reviews/medication discharge summaries performed and professional confidence may reflect the pedagogic principles for advanced education. Early learning means gaining knowledge of facts, whereas advanced learning requires understanding, application, and analyses in order to provide a basis for synthesizing and application of knowledge under new circumstances [22]. Indeed, the weak correlation found between factual drug knowledge and treatment appropriateness [23] illustrates that the basics of pharmacotherapy in real life need to be taught and trained during medical school.

Integrating clinical pharmacology in the internal medicine course may be favorable when aiming to improve prescribing skills in medical students, as medicines are key treatment options for internists. This integration could also facilitate engagement of colleagues and tutors during the ward-based education. Further, clear learning outcomes, focusing on improving students’ practical skills, may increase the attendance in educational activities, thereby increasing the probability of students achieving a sufficient level of knowledge [24]. However, the fact that our clinical pharmacology course is not examined separately in the written examination may affect the level of knowledge achieved by the students [13].

Knowledge of the pharmacology of common drugs is a prerequisite for rational and safe prescribing. In their first clinical year, the medical students encounter a large and sometimes overwhelming number of drugs. The distribution of a list of common drugs could serve as a means to guide the students and help them build a knowledge base in pharmacotherapy. The medication list used here was for self-completion to incorporate active learning and thereby further stimulate learning and students’ performances [25].

Students learn from each other when working in groups and in clinical situations [9, 26–28]. We aimed to stimulate such learning by instructing the students to work together in groups, discussing patient cases during ward attendance, and preparing a presentation on the topic. At the concluding seminar, we experienced that the students’ presentations were generally of high quality and medically relevant. Several groups briefly summarized one or more patient cases as a starting point for a discussion on a specific pharmacotherapeutic topic such as the importance of the renal function, the communication within the team and with the patient, and complex assessments of the benefit-risk balance for drug treatment, for example regarding anticoagulants. Others focused on one case and discussed challenges regarding the diagnosing and treatment of that particular patient, including ethical aspects attached to the choice of treatment.

The results of our study suggest that there may be room for further improvements regarding the acquisition of prescribing knowledge and skills during medical school. After we had modified the course, almost half of the students were not confident in performing medication reviews, and one in five was not confident in writing medication discharge summaries. Further, one in three students had still only performed 0–3 medication reviews, and one in ten only 0–3 medication discharge summaries. In addition, during the ward-based education, only half of the students reflected on whether adverse reactions could have caused a patient’s symptoms, or on the importance of renal function for drug treatment, and even fewer reflected on dosing and drug-drug interactions. As prescribing is a core element within the professional responsibility of a physician, medical schools need to aim at making all students feel confident in managing drug treatment in clinical practice.

A strength of this study is the high response rate, suggesting that the external validity may be acceptable. However, the study was performed in one site only and pre-existing circumstances may differ between medical schools. This may reduce the generalizability of the results. For example, medical schools in many European countries do not provide students with the opportunity to practice prescribing [5]. Nevertheless, our results may be of value in the ongoing efforts to improve clinical pharmacology in undergraduate education [1, 4, 5]. Despite the high response rates, selection bias to some extent cannot be excluded as all students did not respond to the questionnaire.

A limitation of our study is that we used historical controls for the comparison. Therefore, inferences of causality must be interpreted with caution. It may also be argued that students who are already confident in prescribing perform more medication reviews/discharge summaries than students who feel less confident. In addition, self-perceived confidence is not an ideal outcome; it correlates significantly but weakly with prescribing competence [29]. Further, as students do not have a license to prescribe, the perceived confidence may differ from that experienced under postgraduate conditions when the physician actually shoulders the drug treatment responsibility. Indeed, medical students in Sweden can prepare medication reviews/discharge summaries but the responsible ward physician is the one to make the prescribing decisions. Further research is needed to evaluate objective effects.

In conclusion, this study shows that a modified course in clinical pharmacology during medical school, focusing on clear learning outcomes and students’ training during their ward-based education, was associated with increased professional confidence in the basics within the art of prescribing that is to perform medication reviews and to write medication discharge summaries.

## Electronic supplementary material

ESM 1 (PDF 76 kb)

ESM 2 (PDF 96 kb)

ESM 3 (PDF 126 kb)

ESM 4 (PDF 95 kb)
