# Supplementary material for: Developing confidence in basic prescribing skills during medical school: a longitudinal questionnaire study investigating the effects of a modified clinical pharmacology course
Source: Eur J Clin Pharmacol. 2018 Jun 28;74(10):1343–9. doi: 10.1007/s00228-018-2508-3 (PMC6132548; doi:10.1007/s00228-018-2508-3)
Supplement: Supplementary file 1 — (PDF 76 kb) [file 228_2018_2508_MOESM1_ESM.pdf]

Learning outcomes for clinical training of drug prescribing during ward-based education

Knowledge and understanding

- Demonstrate knowledge of drugs which are commonly used in internal medicine, and suggest appropriate pharmacological treatment in uncomplicated clinical situations

Skills and abilities

- Demonstrate ability to perform a medication review, including collection of all relevant information regarding the patient's present treatment and assessment of the appropriateness of the treatment given the current health status of the patient.
- Demonstrate the ability to write a medication discharge summary and actively participate in the information transfer to the patient and his/her relatives
