# Supplementary material for: Developing confidence in basic prescribing skills during medical school: a longitudinal questionnaire study investigating the effects of a modified clinical pharmacology course
Source: Eur J Clin Pharmacol. 2018 Jun 28;74(10):1343–9. doi: 10.1007/s00228-018-2508-3 (PMC6132548; doi:10.1007/s00228-018-2508-3)
Supplement: Supplementary file 2 — (PDF 96 kb) [file 228_2018_2508_MOESM2_ESM.pdf]

List of commonly prescribed drugs

We have compiled this list to facilitate the learning of commonly prescribed drugs. Fill out the list, the first substance on the list (salbutamol) serves as an example. Information can be found for instance in FASS (the Swedish National Formulary) and in REK (the regional prescribing guidelines booklet). Mark with an X in the last column if the substance is recommended in REK.

Allergy/Pulmonary

Substance	Trade name(s)	Mechanism of action/substance group	Indication(s)	Recommended dosing	REK
salbutamol	Buventol, Ventilastin etc	Short acting ß$_2$-receptor agonist	Asthma, COPD	100-200 µg	x
terbutaline					
budesonide					
formoterol					
salmeterol					
indacaterol					
olodaterol					
budesonide + formoterol					
salmeterol + fluticasone					
montelukast					
ipratropium					
tiotropium					
glyko-pyrronium					

indakaterol+ glykopyrron					
olodaterol + tiotropium					
acetylcysteine					
desloratadine					
loratadine					

Blood

Substance	Trade Name	Mechanism of action/ Substance group	Indication(s)	Recommended dosing	REK
dalteparin					
tinzaparin					
enoxaparin					
cyanocobalamin					
folic acid					
ferrous sulfate					

Diabetes					
Substance	Trade Name	Mechanism of action/Substance group	Indication(s)	Recommended dosing	REK
metformin					
glimepiride					
liraglutide					
sitagliptin					
dapagliflozin					
empagliflozin					
insulin	Read the text in the REK booklet				
orlistat					

Endocrinology					
Substance	Trade Name	Mechanism of action/Substance group	Indication(s)	Recommended dosing	REK
betamethasone					
prednisolone					
hydrocortisone					
levothyroxine					
cholecalciferol					

Cardiovascular					
Substance	Trade Name	Mechanism of action/Substance group	Indication(s)	Recommended dosing	REK
enalapril					
ramipril					
hydrochlorthiazide					
hydrochlorthiazide + amiloride					
bendroflumethiazide					
candesartan					
losartan					
metoprolol					
bisoprolol					
atenolol					
amlodipine					
felodipine					
simvastatin					
atorvastatin					
rosuvastatin					
ezetrol					

acetylsalicylic acid (ASA, low dose)					
clopidogrel					
ticagrelor					
glyceroltrinitrat					
isosorbide mononitrate					
furosemide					
spironolactone					
eplerenone					
warfarin					
apixaban					
rivaroxaban					
dabigatran					
digoxin					
dipyridamole					
dipyridamole + ASA					

Gastrointestinal

Substance	Trade Name	Mechanism of action/Substance group	Indication(s)	Recommended dosing	REK
lactulose					

sterculia					
sodium picosulfate					
macrogol					
loperamide					
omeprazole					
esomeprazole					
mesalazine					

Osteoporosis

Substance	Trade Name	Mechanism of action/Substance group	Indication(s)	Recommended dosing	REK
alendronic acid					
calcium + vitamin D					

Rheumatology

Substance	Trade Name	Mechanism of action/Substance group	Indication(s)	Recommended dosing	REK
allopurinol					
methotrexate					

Pain

Substance	Trade Name	Mechanism of action/Substance group	Indication(s)	Recommended dosing	REK
paracetamol					
naproxen					
ibuprofen					
diclofenac					
ketoprofen					
celecoxib					
etoricoxib					
morphine (short acting)					
morphine (long acting)					
oxicodone (short acting)					
oxicodone (long acting)					
fentanyl					
codeine + paracetamol					
tramadol					
amitriptyline					
nortriptyline					

gabapentin					
pregabalin					

Neurology					
Substans	Trade Name	Mechanism of action/Substance group	Indication(s)	Recommended dosing	REK
lamotrigine					
carbamazepine					
valproic acid					
levetiracetam					

Psychiatry					
Substance	Trade Name	Mechanism of action/Substance group	Indication(s)	Recommended dosing	REK
sertraline					
citalopram					
escitalopram					
fluoxetine					
paroxetine					
mirtazapine					

venlafaxine					
	Cymbalta				
lithium					
oxazepam					
diazepam					
alprazolam					
zopiclone					
zolpidem					
propiomazine					
alimemazine					
hydroxyzine					
prometazine					
olanzapine					
aripiprazole					
risperidone					
donepezil					
memantine					
methylphenidate					

Urology					
Substance	Trade Name	Mechanism of action/substance group	Indication(s)	Recommended dosing	REK
alfuzosin					
finasteride					
bicalutamide					
tolterodine					
