# Supplementary material for: Developing confidence in basic prescribing skills during medical school: a longitudinal questionnaire study investigating the effects of a modified clinical pharmacology course
Source: Eur J Clin Pharmacol. 2018 Jun 28;74(10):1343–9. doi: 10.1007/s00228-018-2508-3 (PMC6132548; doi:10.1007/s00228-018-2508-3)
Supplement: Supplementary file 3 — (PDF 126 kb) [file 228_2018_2508_MOESM3_ESM.pdf]

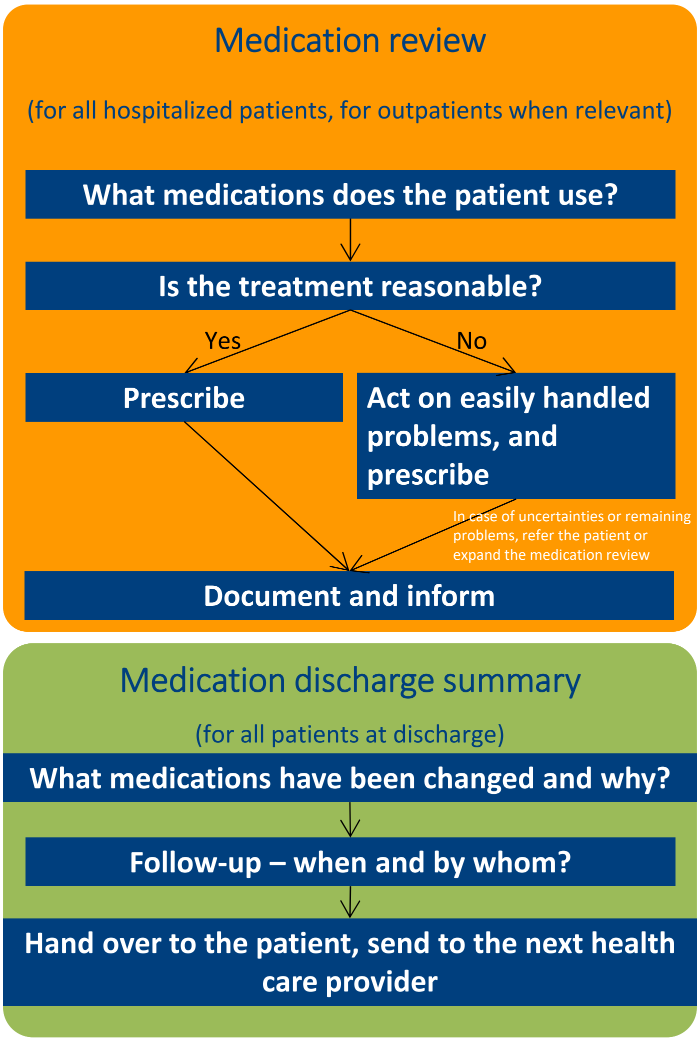
Medication review
(for all hospitalized patients, for outpatients when relevant)
What medications does the patient use?
Is the treatment reasonable?
Yes
No
Prescribe
Act on easily handled problems, and prescribe
In case of uncertainties or remaining problems, refer the patient or expand the medication review
Document and inform
Medication discharge summary
(for all patients at discharge)
What medications have been changed and why?
Follow-up – when and by whom?
Hand over to the patient, send to the next health care provider

What medications does the patient use?

Sources:
- Patient/relatives
- Medical record
- Multi-dose drug dispensing system
- Dispensed Drug Register
- Medication lists from other health care providers

[Instructions specific for the medical record system, including keyboard shortcuts]

[Useful shortcuts in the medical records]

Is the treatment reasonable?

Assess from medical knowledge and informed common sense. Use the integrated alert tool to check for potential interactions and double medications. Pay attention to the renal function, use the integrated renal function calculator. Adjust and prescribe.

Document and inform

Report source/s/ used for reconciliation and comment on essential findings or changes. Inform and provide the patient with an updated medication list. Ascertain that the medications in the medical record are updated.

What medications have been changed and why?

Describe the drug treatment provided during hospitalization and the changes made, including reasoning

Follow-up – when and by whom?

Report planned follow-up (when and by whom)

Hand over to the patient, send to the next health care provider

Inform and hand over to the patient, together with an updated medication list. Send to the next health care provider.
