# Supplementary material for: Developing confidence in basic prescribing skills during medical school: a longitudinal questionnaire study investigating the effects of a modified clinical pharmacology course
Source: Eur J Clin Pharmacol. 2018 Jun 28;74(10):1343–9. doi: 10.1007/s00228-018-2508-3 (PMC6132548; doi:10.1007/s00228-018-2508-3)
Supplement: Supplementary file 4 — (PDF 95 kb) [file 228_2018_2508_MOESM4_ESM.pdf]

A questionnaire on drug treatment and learning

- WBE = Ward-Based Education

Please mark to what extent you agree with the following statements: (on a scale from "Strongly disagree to Strongly agree")	*Strongly disagree*				*Strongly agree*
**1**. During WBE, I usually reflected on what medications my patient was ordered	☐	☐	☐	☐	☐
**2**. During WBE, I usually reflected on what medications my patient was actually using	☐	☐	☐	☐	☐
**3**. During WBE, I usually reflected on whether my patient's drug treatment was reasonable given his/her present condition	☐	☐	☐	☐	☐
**4.** During WBE, I usually considered adverse effects as a possible cause of symptoms	☐	☐	☐	☐	☐
**5.** During WBE, I usually assessed solely the medications related to the medical situation causing the present hospitalisation	☐	☐	☐	☐	☐
**6**. During WBE, I usually reflected on preventing duplicate medications (e.g. two different paracetamol drugs)	☐	☐	☐	☐	☐
**7.** During WBE, I usually reflected on whether the dose was reasonable	☐	☐	☐	☐	☐
**8.** During WBE, I usually reflected on the importance of the patient's kidney function for the drug treatment	☐	☐	☐	☐	☐
**9.** During WBL, I usually reflected on whether there were drug-drug interactions	☐	☐	☐	☐	☐
**10.** During WBE, I used to check for drug interactions using the computerized decision support system embedded in the electronic medical record	☐	☐	☐	☐	☐
**11.** During WBE, I used to check for drug interactions using an external drug interaction tool NOT embedded in the medical record	☐	☐	☐	☐	☐
**12.** When a drug was discontinued, I usually reflected on that this had to be appropriately followed-up	☐	☐	☐	☐	☐
**13.** At the discharge of "my" patient, I used to reflect on follow-up of his/her drug treatment	☐	☐	☐	☐	☐
**14**. I know the components of a medication review	☐	☐	☐	☐	☐
**15.** I feel confident in performing a medication review	☐	☐	☐	☐	☐

	Strongly disagree				*Strongly agree*
**16**. I know the components of a medication discharge summary	☐	☐	☐	☐	☐
**17.** I feel confident in writing a medication discharge summary	☐	☐	☐	☐	☐
**18**. I feel confident with the prescription writing standards (paper or electronic) for a prescription to be filled in a pharmacy	☐	☐	☐	☐	☐
**19**. I feel confident with writing standards for medication orders during inpatient care, within the electronic medical record	☐	☐	☐	☐	☐
**20.** I reckon that I, before WBE, had acquired sufficient knowledge regarding mechanisms of action, effects, and adverse effects of drugs	☐	☐	☐	☐	☐
**21.** I reckon that I, before WBE, had acquired sufficient knowledge regarding the principles of pharmacokinetics	☐	☐	☐	☐	☐
**22**. The knowledge of principles of drug treatment that I have acquired during my studies is sufficient to serve as a basis for my training during WBE	☐	☐	☐	☐	☐
**23**. During WBE, I have been supervised in my clinical training within pharmacotherapy by a clinician	☐	☐	☐	☐	☐
**24**. During WBE, I have received feed-back from a supervisor/clinician regarding my clinical training within pharmacotherapy	☐	☐	☐	☐	☐

25. During WBE, I contributed to ………………………. medication reviews
(approximate number)

26. During WBE, I contributed to ………………………. medication discharge summaries
(approximate number)

27. During WBE I used the following sources to find information about a patient's prescribed medications (Mark all boxes that apply)

☐ The patient ☐ Register of dispensed drug ☐ Multi-dose dispensing system

☐ Medical records ☐ Pharmacy register of current prescriptions available for dispensing

☐ Other…………………………………………………………..

28. I am …………………………..years old

29. I am a: ☐ woman ☐ man

30. I: ☐ have never been involved in research

☐ am/have been involved in research but I am not a PhD

☐ am a PhD

Comments and reflections on teaching and learning in pharmacology and clinical pharmacology:

…………………………………………………………………………………...………………
…………………………………………………………………………………...………………
……..………………………………………………………………………………………
……..………………………………………………………………………………………
……..………………………………………………………………………………………

We thank you for your participation in the survey
